# ASO Author Reflections: What is “Recovery”? Lessons from Patient-Reported Outcomes in Gastrectomy

**DOI:** 10.1245/s10434-026-19891-9

**Published:** 2026-06-03

**Authors:** Maho Takayama, Naruhiko Ikoma

**Affiliations:** Department of Surgical Oncology, The University of Texas MD Anderson Cancer Center, Houston, TX USA

## Past

“How long will it take to recover from surgery?” Although this is one of the most common questions patients ask, surgeons rarely have a precise answer. The fundamental problem is that “recovery” itself has never been clearly defined. In gastrectomy, this question is especially important because gastric surgery directly affects appetite, eating, and daily functioning—core components of quality of life. Yet postoperative recovery has traditionally been inferred from surrogate markers such as complications, length of stay, or readmission, rather than measured from the patient perspective.

## Present

In the current study, we used the upper gastrointestinal module of the MD Anderson Symptom Inventory (MDASI)^1,2^ to longitudinally characterize postoperative symptoms and functional interference after gastrectomy. This allowed us to define patient-reported recovery objectively, in terms of improvement of both symptom severity and functional interference from their postoperative peak.^3^ Using this framework, we showed that recovery trajectories differ substantially across gastrectomy procedures in both tempo and pattern. Patients undergoing total gastrectomy showed delayed recovery compared with other procedures, with only 75% recovering by postoperative month 6.

However, postoperative recovery after gastrectomy cannot be fully explained by procedure type alone.^4^ Recovery is likely shaped by multiple factors, including operative approach, reconstruction, and postoperative symptom patterns. Therefore, a standardized and patient-centered definition of recovery—such as the framework proposed in the current study—will be essential for future comparisons of gastrectomy procedures and perioperative care.

## Future

We are further developing a streamlined symptom-monitoring platform derived from selected MDASI items to facilitate practical assessment of recovery in routine clinical care.^5^ Such tools may help surgeons provide patients with realistic expectations regarding postoperative recovery while offering a standardized benchmark for tracking surgical outcomes. Beyond defining when patients recover, future work should also more deeply explore how recovery differs among procedures, including procedure-specific symptom trajectories after total versus proximal gastrectomy. Defining recovery through patient-reported outcomes may ultimately shift postoperative evaluation from surgeon-centered metrics toward a more patient-centered understanding of surgical success.
